# Beyond neuronal loss: epigenetic signatures bridging immune activation and sleep dysfunction in narcolepsy type 1

**DOI:** 10.1093/sleep/zsaf253

**Published:** 2025-08-23

**Authors:** Ahmed S BaHammam

**Affiliations:** University Sleep Disorders Center, Department of Medicine, College of Medicine, King Saud University, Riyadh, Saudi Arabia

**Keywords:** narcolepsy type 1, epigenetics, DNA methylation, T-cell immunology, biomarkers

The field of sleep medicine finds itself at a crossroads, where traditional boundaries between neurology, immunology, and genetics are being redefined by the emergence of the “epigenome” as a key nexus for disease pathogenesis. Nowhere has this been more apparent than in narcolepsy type 1 (NT1), a disorder long recognized for its distinctive clinical profile and Human Leukocyte Antigen (HLA) associations, but equally notorious for being resistant to simple mechanistic explanations. In this context, the paper by Shimada *et al.* signals a timely expansion of our conceptual framework [1]. This work demonstrates how integrated multi-omics and cell-type-specific epigenetic profiling can illuminate mechanisms that decades of candidate gene and Genome-Wide Association Studies (GWAS) approaches could not, pointing toward a dynamic molecular interface between environmental exposures, immune dysregulation, and the neuronal vulnerability characteristic of NT1.

Epigenetics refers to heritable changes in gene expression that occur without alterations to the underlying DNA sequence. These changes, which can be maintained through cell divisions and sometimes across generations, are primarily mediated by DNA methylation and histone modifications [2]. DNA methylation, involving the addition of methyl groups to cytosine residues, particularly in promoter regions, typically represses gene expression. Together, these epigenetic mechanisms form a dynamic regulatory system that can respond to environmental stimuli, developmental cues, and disease states.

## Moving beyond genetic predisposition to a layered etiology

For more than twenty years, research into NT1 has been characterized by its robust genetic associations, above all, the exceptional prevalence of the HLA-DQB1*06:02 allele in patients [3]. However, this allele is neither necessary nor sufficient for phenoconversion. Rather, it sets the stage for a more complex interplay of genetic and environmental risk factors, including viral exposures and, in rare cases, immune adjuvants [3]. Genome-wide association studies have implicated T-cell receptor loci [4]. Building on these findings, targeted genetic studies using immune-focused arrays have further identified additional immune-regulatory genes, including Tumor Necrosis Factor Superfamily Member 4 (TNFSF4) and Cathepsin H [5]. These findings consistently point to immune-mediated pathogenesis.

While such genetic signals are undeniably important, the translation from association to causality has proven elusive, given the relatively modest overall heritability [6]. Therefore, research has shifted toward identifying regulatory and cell-type-restricted mechanisms through which these susceptibility alleles interact with environmental triggers. Epigenetic studies, with their ability to capture such gene–environment interactions, are therefore uniquely positioned to advance the field of narcolepsy research. These converging mechanisms are summarized in Table 1, outlining the evolving pathophysiological framework of NT1 from genetic predisposition to potential therapeutic opportunities.

**Table 1:** Integrative pathophysiological framework of narcolepsy type 1: key mechanisms linking genetic risk, environmental triggers, immune dysregulation, and epigenetic alterations, as contextualized by Shimada *et al.* (2025) and prior research

**Conceptual layer**	**Description**	**Key insights/findings**
**Genetic susceptibility**	Predisposing alleles increase NT1 risk	>90% of NT1 patients carry HLA-DQB1*06:02; GWAS implicate TCR loci and immune genes
**Environmental triggers**	External exposures initiate an immune response	Onset often follows H1N1 influenza, infections, or immune adjuvants
**Hypothalamic neuron involvement**	Orexin-producing neurons are lost or transcriptionally silenced	Central hypocretin deficiency underlies core symptoms
**Peripheral T Cell dysregulation**	CD4^+^/CD8^+^ T cells show altered function and phenotypes	Autoreactive T cells target hypocretin-related antigens
**Epigenetic modifications**	DNA methylation regulates gene expression without altering the sequence	NT1-associated global hypomethylation in T cells; non-promoter CpG regions affected
**Chemokine signaling pathways**	Guide immune cell migration and activation	Altered regulation of CCL5, CCR4, S100A4; linked to clonal proliferation
**Multi-omics integration**	Merges methylome, transcriptome, and genotype data	Identifies robust, replicable biomarkers, and pathways relevant to NT1
**Therapeutic implications**	Potential targets for biomarker development and immune-epigenetic therapies	Potential for chemokine modulation, HDAC inhibitors, and DNA methyltransferase inhibitors

Abbreviations**:** NT1, narcolepsy type 1; HLA, human leukocyte antigen; GWAS, genome-wide association study; TCR, T-cell receptor; H1N1, hemagglutinin type 1/neuraminidase type 1 (influenza subtype); CD, cluster of differentiation; CpG, cytosine-phosphate-guanine; CCL5, C-C motif chemokine ligand 5; CCR4, C-C motif chemokine receptor 4; S100A4, S100 calcium-binding protein A4; HDAC, histone deacetylase.

## DNA methylation: mechanistic elegance amidst biological complexity

The study by Shimada *et al.* stands out for its rigorous design and innovative use of sorted peripheral T cell subsets to interrogate disease-associated DNA methylation patterns, transcriptomics, and genotype data, all within the same subjects [1]. This strategy circumvents the dilution and phenotypic heterogeneity that have plagued earlier methylome studies using bulk blood or brain samples [7, 8].

To fully contextualize these peripheral immune findings, it is essential to consider parallel epigenetic phenomena occurring within the central nervous system. Recent work by Seifinejad *et al.* provides evidence that, in NT1, the loss of orexin (hypocretin) function may stem from epigenetic silencing of the orexin gene itself, rather than from autoimmune destruction of orexin-producing neurons [9]. Their analysis of hypothalamic tissue from patients with narcolepsy revealed hypermethylation at the promoter region of the orexin gene, supporting a model in which transcriptional repression, rather than overt neuronal loss, accounts for the observed hypocretin deficiency [9].

By focusing on purified CD4+ and CD8+ T cells, which are increasingly implicated in immune surveillance, autoimmunity, and even neurodegeneration, Shimada *et al.* have not only recapitulated known immune signatures of NT1 [1, 10, 11], but also identified a broad, reproducible pattern of global hypomethylation. This epigenetic signature is most pronounced in non-promoter, non-CpG island regions, echoing similar findings in neurodegenerative and autoimmune diseases [12, 13].

The global hypomethylation observed was not random; it clustered within genomic regions encoding key chemokines and chemokine receptors such as CCL5, CCR4, and S100A4, and was correlated with proliferation-associated hypomethylation indices *(“hypoSC” index, which reflects mitotic activity estimated from methylation profiles*) [1]. The data suggest an ongoing state of immune activation and T cell clonal proliferation, aligning with evidence from flow cytometry and functional studies that T cell memory subsets are dysregulated in NT1 [10].

Furthermore, pathway analyses consistently identified enrichment of genes involved in chemotaxis and cell migration. The S100A4-associated differentially methylated position was particularly remarkable for its multi-omics support through methylation quantitative trait locus (mQTL) analysis (genetic variants that influence DNA methylation levels at nearby sites). Through integrative analysis, the authors inferred that S100A4 expression is likely elevated in CD4+ T cells from patients with NT1 due to hypomethylation. This calcium-binding protein (S100A4) regulates cellular motility and chemotaxis, and has been implicated in promoting T-cell migration [14].

## Integrative omics: reducing false positives, increasing biological plausibility

A key contribution of Shimada *et al.*’s study, beyond the technical execution, is its integrative analytic framework [1]. By prioritizing regions of differentially methylated regions (DMRs) over single-site differentially methylated positions (DMPs), and by requiring replication and corroboration across methylome, transcriptome, and genotype data, the investigators have set a new bar for rigor in the field. This “triangulation” approach drastically reduces the likelihood of false-positive findings driven by batch effects, array artifacts, or minor population stratification.

Weighted Gene Co-expression Network Analysis (WGCNA) analysis identified only one statistically significant association after multiple testing correction (MEivory module with sex). Two additional modules showed nominal associations with sleep parameters (rapid eye movement (REM)-sleep latency by polysomnography and average-sleep latency by multiple sleep latency test), but did not survive correction. These preliminary findings suggest a possible link between immune-related gene expression and NT1 phenotypic variation, but do not provide strong evidence for molecular subtyping.

## Implications for biomarker development and future therapy

If we consider NT1 as the paradigmatic “multi-hit disorder,” this study demonstrates how complex disease biology may be deconvoluted at the level of immune cell epigenetics. From a translational standpoint, several key implications emerge:

- **Diagnostic enrichment:** Hypomethylation patterns in peripheral T cells may complement established biomarkers such as cerebrospinal fluid (CSF) orexin-A and HLA genotyping, offering less invasive options for earlier diagnosis and potential clinical subtyping [2, 15].
- **Mechanistic stratification:** By connecting chemotactic and proliferative signatures to immune dysregulation in NT1, Shimada *et al.* suggest potential avenues for future patient stratification. Clinicians may one day move beyond “one-size-fits-all” management, instead targeting therapies (e.g. modulation of chemokine pathways) to molecularly-defined subgroups [1]. The broader precedent for using histone deacetylase inhibitors in neurodegenerative disorders and DNA methyltransferase inhibitors in oncology and autoimmunity highlights the translational potential of epigenetic modulation in complex disease contexts [16, 17].
- **Treatment monitoring and drug development:** The relative stability of these methylation profiles suggests they could serve as future biomarkers for monitoring treatment response or relapse, and may also inform the development of epigenetic therapies. Nonetheless, extensive validation is required.
- **Epigenetic Therapeutic Horizons**: The clinical success of histone deacetylase inhibitors (e.g. vorinostat in T-cell lymphoma) and DNA methyltransferase inhibitors in oncology, together with emerging investigations of these agents in autoimmune and neurodegenerative disorders, underscores the translational potential of epigenetic modulation for complex neurological diseases such as NT1 [16–18].

## Current limitations, unanswered questions, and future directions

While Shimada *et al.* provide robust cross-sectional evidence, several critical questions remain. How stable are these T cell methylation patterns across the natural history of NT1? Do they fluctuate with disease activity, treatment, or remission? Addressing these issues will require longitudinal studies, ideally integrating paired blood and CSF biomarker profiles. Ultimately, it is imperative to distinguish whether the observed epigenetic alterations represent causal mechanisms or secondary consequences of immune dysregulation.

Several methodological considerations from Shimada *et al.*’s work warrant careful attention for future investigations. The study’s exclusive focus on a Japanese population, while providing genetic homogeneity advantageous for HLA-linked disease mechanisms, necessitates validation across ethnically diverse cohorts to establish broader clinical relevance. Additionally, the application of methylation-derived protein indicators, originally developed for whole blood analyses, to isolated T cell populations may introduce technical uncertainties that necessitate the development of cell-type-specific validation approaches. The relatively modest sample sizes, although reasonable for this rare condition, limited statistical power for detecting subtle epigenetic variations and precluded comprehensive multiple testing corrections beyond the findings in the HLA region. Furthermore, the use of bulk RNA sequencing from whole blood rather than matched isolated T cell populations may have potentially obscured cell-specific transcriptional changes; however, deconvolution analyses suggested minimal confounding from differences in cellular composition between cases and controls.

Finally, emerging single-cell multi-omics and spatial epigenetics technologies hold promise for refining and extending these findings, enabling the construction of a more detailed pathophysiological “atlas” of narcolepsy.

## Conclusion

The findings of Shimada et al. mark a significant advance in our understanding of NT1, not only by identifying replicable disease-associated methylation changes in functionally relevant T cell subsets, but also by providing a roadmap for the application of integrative multi-omics to complex neurological disorders [1]. The groundwork laid by this paper will inform both biomarker development and mechanistic modeling, shaping the next wave of personalized interventions for patients with narcolepsy.
